# Comparison of automated quantification of amyloid deposition between PMOD and Heuron

**DOI:** 10.1038/s41598-023-36986-5

**Published:** 2023-06-19

**Authors:** Hyun Woong Roh, Sang Joon Son, Chang Hyung Hong, So Young Moon, Sun Min Lee, Sang Won Seo, Seong Hye Choi, Eun-Joo Kim, Soo Hyun Cho, Byeong Chae Kim, Seongbeom Park, Soohwa Song, Young-Sil An

**Affiliations:** 1 Department of Psychiatry, School of Medicine, Ajou University, Suwon, Korea; 2 Department of Neurology, School of Medicine, Ajou University, Suwon, Korea; 3 Department of Neurology, Samsung Medical Center, School of Medicine, Sungkyunkwan University, Seoul, Korea; 4 Department of Neurology, School of Medicine, Inha University, Incheon, Korea; 5 Department of Neurology, Pusan National University Hospital, Medical Research Institute, Pusan National University School of Medicine, Busan, Korea; 6 Department of Neurology, Chonnam National University Medical School, Chonnam National University Hospital, Gwangju, Korea; 7 Heuron Co., Ltd., Incheon, Korea; 8 Department of Nuclear Medicine and Molecular Imaging, School of Medicine, Ajou University, 206, World cup-ro, Yeongtong-gu, Suwon-si, Gyeonggi-do, Suwon, 16499 Korea

**Keywords:** Psychology, Neurology

## Abstract

Several programs are widely used for clinical and research purposes to automatically quantify the degree of amyloid deposition in the brain using positron emission tomography (PET) images. Given that very few studies have investigated the use of Heuron, a PET image quantification software approved for clinical use, this study aimed to compare amyloid deposition values quantified from ^18^F-flutemetamol PET images using PMOD and Heuron. Amyloid PET data obtained from 408 patients were analysed using each quantitative program; moreover, the standardized uptake value ratios (SUVRs) of target areas were obtained by dividing the standardized uptake value (SUV) of the target region by the SUV of cerebellar grey matter as a reference. Compared with PMOD, Heuron yielded significantly higher SUVRs for all target areas (paired sample t-test, *p* < 0.001), except for the PC/PCC (*p* = 0.986). However, the Bland–Altman plot analysis indicated that the two quantitative methods may be used interchangeably. Moreover, receiver operating characteristic curve analysis revealed no significant between-method difference in the performance of the SUVRs in evaluating the visual positivity of amyloid deposits (*p* = 0.948). In conclusion, Heuron and PMOD have comparable performance in quantifying the degree of amyloid deposits in PET images.

## Introduction

Alzheimer’s disease (AD) is the most common cause of dementia, accounting for > 80% of dementia cases^1^. AD is a neurodegenerative disease that mainly occurs among individuals aged > 65 years. Patients with AD present with cognitive decline and psychiatric symptoms such as personality changes, depression, delusions, increased aggression, and sleep disorders^2^. As the ageing society accelerates, the number of patients with AD is increasing, which is becoming a social and economic issue^3^.

Although there remains no definitive cure for AD, early diagnosis and prompt interventions and care are widely considered to significantly improve the quality of life for patients with AD^4^. The clearest pathological hallmark of AD is β-amyloid plaque deposition and neurofibrillary tangles composed of tau aggregates in the brain^5^. However, the clinical utility of this pathological hallmark is limited by the difficulty of performing invasive biopsies to obtain brain specimens. Nonetheless, position-emission tomography (PET) imaging allows non-invasive assessment of amyloid plaque deposition in the brain and is being clinically used for the differential diagnosis of AD^6^. Initially, radiopharmaceutical labelling in amyloid PET was performed using C-11 Pittsburgh Compound B (PIB), with subsequent application of F-18 (F-18 flutemetamol, F-18 florbetaben, F-18 florapronol, etc.) and commercialisation, which has made it available to institutions without a cyclotron^7^.

The principal method of evaluating amyloid PET images is the visual examination for the presence or absence of amyloid deposits. However, PET scan results are only reliable when obtained by well-trained expert readers^8^ and even then, they may remain ambiguous. There has been increasing interest in complementary quantitative methods for indicating the degree of deposition in amyloid PET^9^. Quantitative analysis of amyloid PET images allows manual selection of the regions of interest (ROIs); however, its clinical utility is impeded by its time consumption and variability across readers. Accordingly, various automatic quantification software programs have been introduced and compared^10–13^ to inform the selection of the optimal software program for each scenario.

Several commercially available software tools can automatically quantify the uptake of radiopharmaceuticals by brain regions, based on amyloid PET images. Among them, several programs such as MIM Software’s *MIMneuro*, GE Healthcare’s *CortexID*, and Siemens Healthineers’ *Syngo.*VIA have been approved for clinical use; however, others such as PMOD, MIAKAT, and CapAIBL are only available for research use^9^. In our institution, PMOD has been used for research for a long time^14,15^. Heuron is a PET image quantification software approved for clinical use; however, as it has only been recently commercialised, few studies have investigated its use.

Therefore, this study aimed to compare the quantification values from PMOD and Heuron, and provide information to those considering their introduction.

## Results

### Visual interpretation of amyloid deposition

A total of 116 (28.4%) and 292 (71.6%) patients exhibited visually positive and negative results, respectively, for amyloid deposition on PET images. The amyloid-positive group was significantly older than the amyloid-negative group (74.3 ± 6.9 vs. 71.1 ± 7.3 years, *p* < 0.001). Regarding the sex distribution of the amyloid-positive group, females accounted for 66.4% (77/116) and males accounted for 33.6% (39/116), and there was no statistically significant difference with the negative group (female/male = 209 (71.6%)/83 (28.4%), *p* = 0.302).

### Comparison of standardized uptake value ratios (SUVRs) by the paired sample t-test

**Figure 1:**
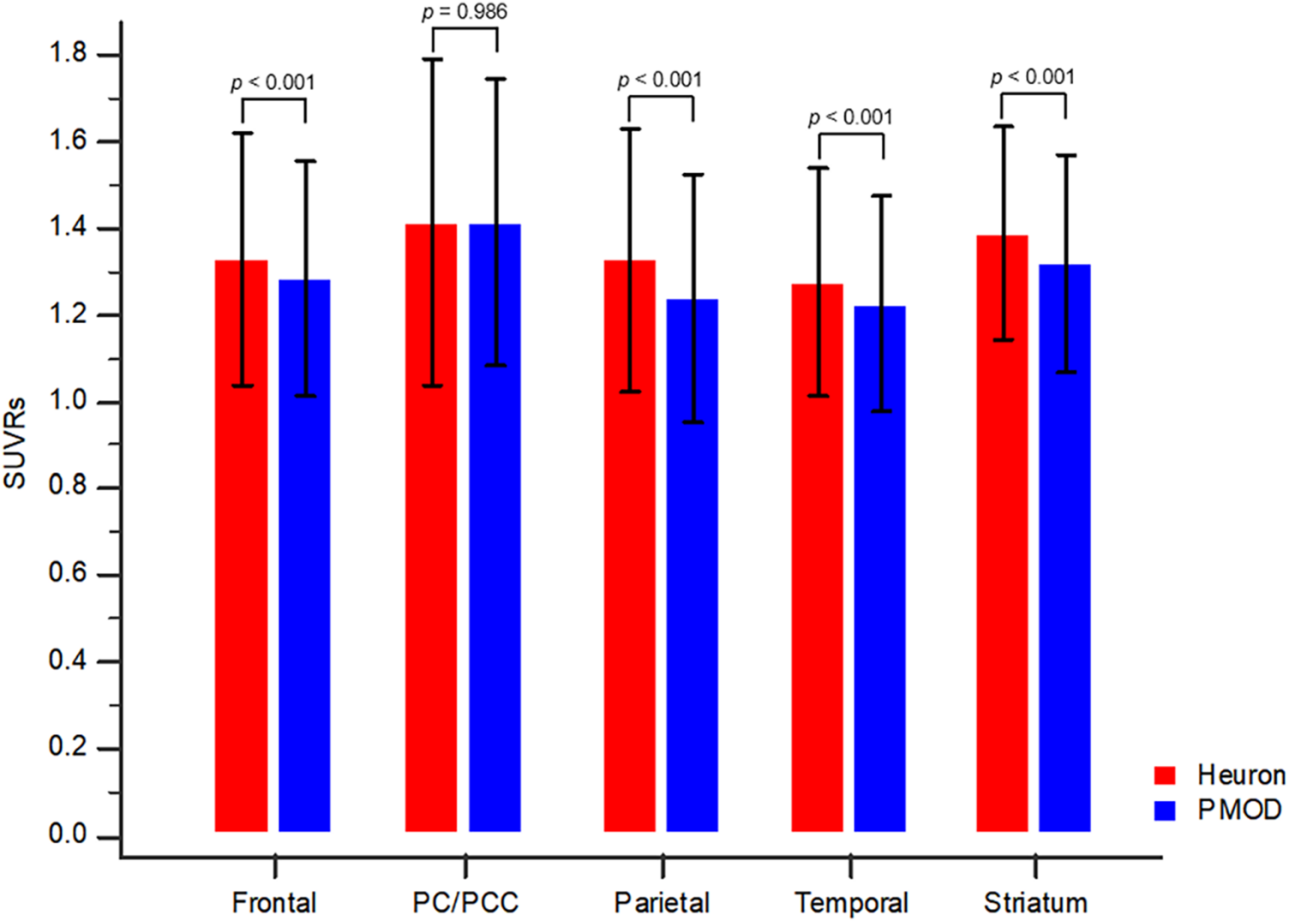
Between-method comparison of standardized uptake value ratios (SUVRs) for brain regions. Compared with PMOD, Heuron yielded significantly higher SUVRs for all target areas, except for the precuneus/posterior cingulate (PC/PCC).

Heuron yielded significantly higher overall SUVRs (including all target regions) than did PMOD (1.35 ± 0.30 vs. 1.30 ± 0.29, *p* < 0.001). Similarly, Heuron yielded significantly higher SUVRs (*p* < 0.001) in each target area, than did PMOD, as follows: frontal lobe (1.32 ± 0.29 vs. 1.28 ± 0.27), parietal lobe (1.33 ± 0.30 vs. 1.24 ± 0.29), temporal lobe (1.27 ± 0.26 vs. 1.22 ± 0.25), and striatum (1.39 ± 0.25 vs. 1.32 ± 0.25). Contrastingly, the SUVRs of the precuneus/posterior cingulate (PC/PCC) region were similar for both quantitative methods (1.41 ± 0.38 vs. 1.41 ± 0.33, *p* = 0.986). Figure 1 shows a graph of the comparison of SUVRs for each target brain area.

### Comparison of SUVRs by the Bland–Altman plot

**Figure 2:**
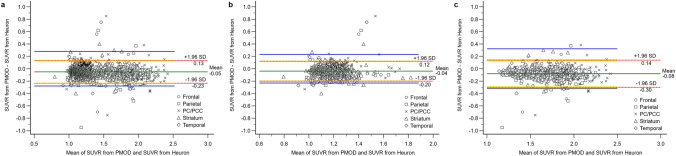
Scatter diagrams of the differences plotted against the averages of the two measurements obtained using PMOD and Heuron. Graph ‘(**a)**’ presents the result from all participants, and the other graphs present the results divided into the visually amyloid-positive (**b**) and amyloid-negative groups (**c**). The mean difference value according to Bland–Altman plot analysis was drawn as a green horizontal line in the graph. The limits of agreement (LoA) representing the mean difference ± 1.96 standard deviations (SD) are indicated by red horizontal dotted lines. The partially yellow lines show the 95% confidence interval of the LoA. Blue horizontal lines represent the maximum allowed between-method difference, which was not exceeded as indicated by the LoAs (red horizontal dotted lines) in all graphs (**a**,**b**,**c**). Therefore, SUVR values yielded by PMOD and Heuron could be used interchangeably.

Based on the Bland–Altman plot analysis, the limits of agreement did not exceed the maximum allowed between-method difference in the overall target areas (Fig. 2a). This pattern was maintained even when the visually negative (Fig. 2b) and positive groups (Fig. 2c) were separately analysed. This indicates that PMOD and Heuron could be used interchangeably in all key brain regions associated with amyloid deposition.

### Diagnostic performance of assessing visual positivity of amyloid deposits

Regarding the SUVR obtained from PMOD, the predictive cutoff value for amyloid deposition positivity was 1.40, with a sensitivity of 94.83% and specificity of 95.89%. It showed excellent performance in distinguishing the amyloid-positive and amyloid-negative groups, with an area under the receiver operating characteristic (ROC) curve (AUC) of 0.989 (*p* < 0.001). Similarly, Heuron showed excellent performance in distinguishing the amyloid-positive and amyloid-negative groups (AUC = 0.988, *p* < 0.001), with a cutoff value of 1.45, sensitivity of 95.69%, and specificity of 95.55%.

**Figure 3:**
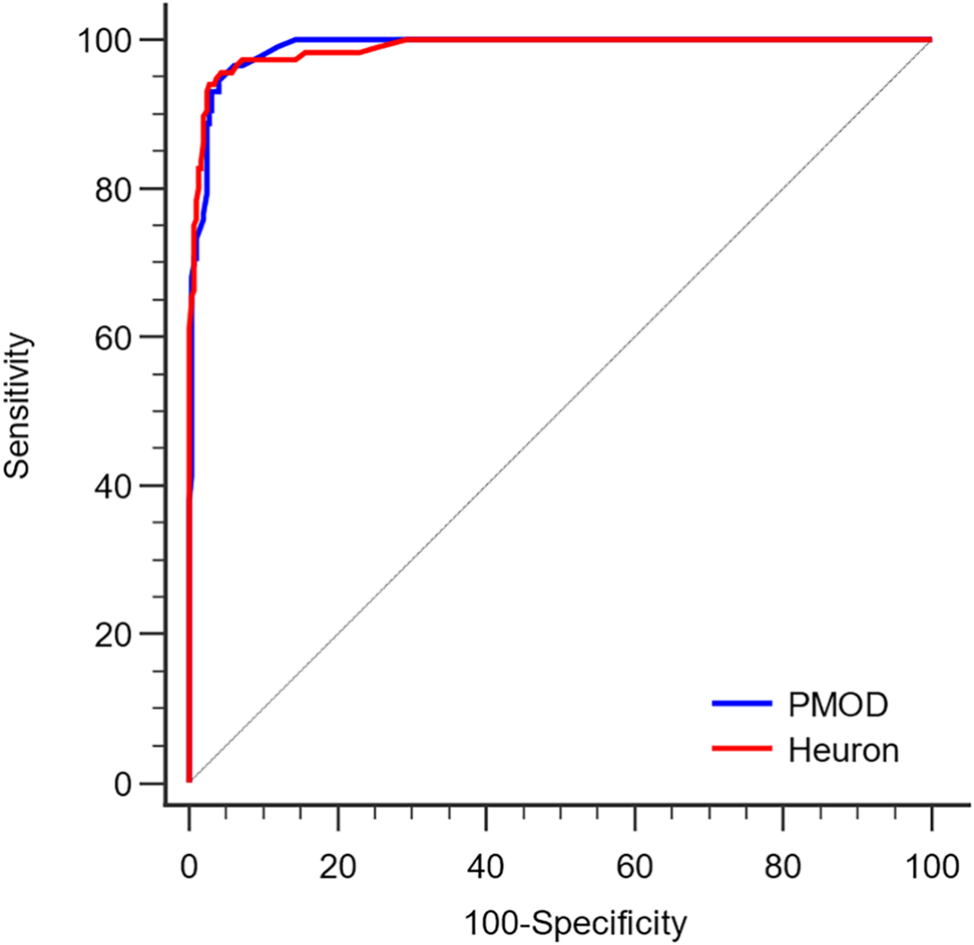
Receiver Operating Characteristic (ROC) curves of SUVR for assessing visual positivity of amyloid deposits. SUVR values obtained using both PMOD and Heuron showed excellent diagnostic performance in detecting the presence or absence of amyloid deposition (area under the ROC curve [AUC] = 0.989 for PMOD and 0.988 for Heuron), with no significant between-group difference (*p* = 0.948).

Between-method comparison of the ROC curves did not reveal significant differences in diagnostic performance (*p* = 0.948). Figure 3 shows the ROC curves for each software tool.

## Discussion

**Figure 4:**
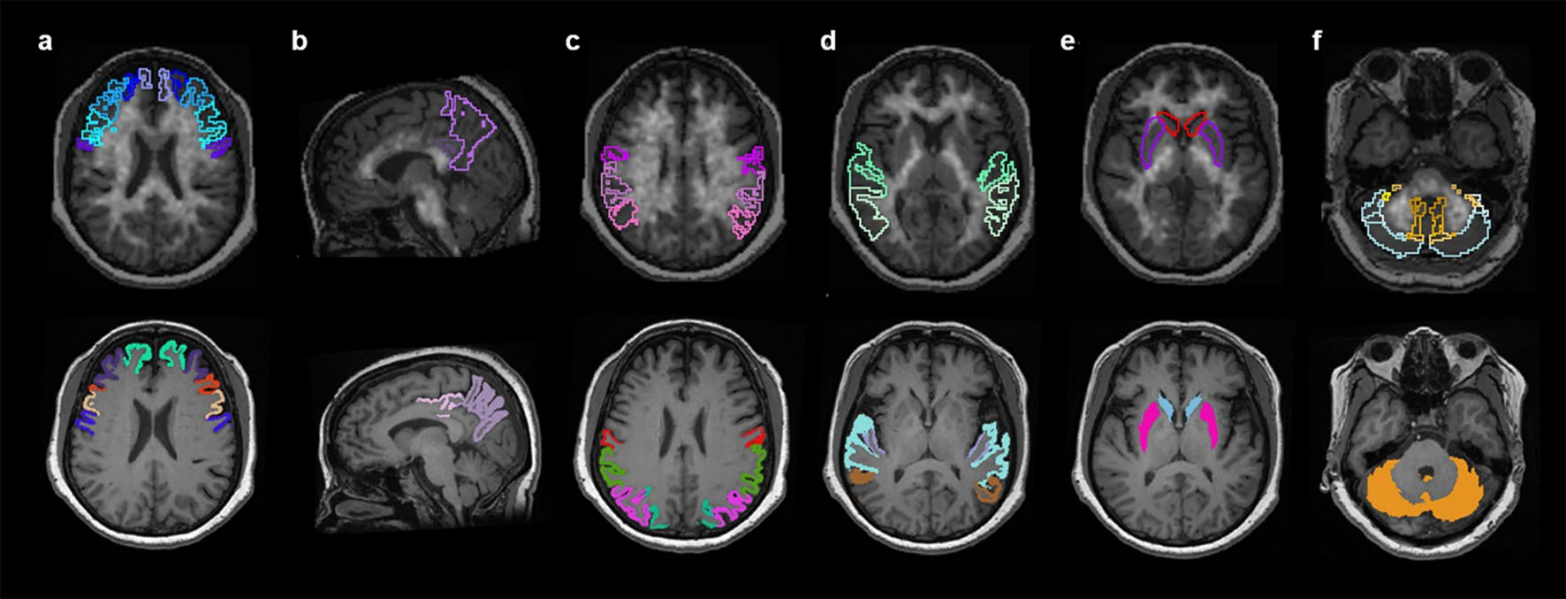
Representative images for contours of volumes of interest (VOIs). The VOIs for the frontal lobe (**a**), precuneus/posterior cingulate (PC/PCC) (**b**), parietal lobe (**c**), temporal lobe (**d**), striatum (**e**), and cerebellar grey matter (**f**) with coloured contours are automatically set in co-registered PET-MR images using the AAL-VOIs atlas provided by PMOD (top row) and MR images fed to Heuron (bottom row).

Our findings indicate that Heuron yielded higher SUVR values than did PMOD in almost all target brain regions, except for the PC/PCC region. This could be attributed to the slight difference in the boundary range when setting the brain area in the two software packages, as shown in Fig. 4. However, we could not conclusively determine whether only the SUVR values of the PC/PCC were similar between the two software packages. Future similar studies are warranted for a clearer interpretation of our results.

Although Heuron presented overall higher SUVR values than did PMOD, Bland–Altman plot analysis indicated that these quantification tools could be used interchangeably. Additionally, ROC curve analysis indicated no significant between-group difference in the diagnostic performance for visual positivity of amyloid deposition; further, the SUVR cutoff values for amyloid deposition positivity were similar between PMOD (1.40) and Heuron (1.45). To our knowledge, this is the first study to compare the quantification values for amyloid PET obtained using Heuron and PMOD. Our findings could inform the selection and implementation of these quantification tools for amyloid PET.

Our predictive cutoff values for visual positivity of amyloid deposition were slightly higher than those obtained in previous similar studies (1.35)^16,17^. This difference could be attributed to our selection of the highest SUVR value in each target region for the ROC curve analysis, while the previous studies used the SUVR of global cerebral regions. Visual confirmation of amyloid deposition in any target brain region on PET images indicates a positive finding. Therefore, even when setting the cutoff value for quantification analysis, we considered the highest SUVR value for each target region as a better value for reflecting the positivity of visual amyloid deposition than the overall SUVR value for all cerebral regions. The cutoff value for amyloid PET positivity may differ depending on the type of radiopharmaceutical used, reference area, analysis tool, etc., which is a practical limitation of applying a uniform value across institutions. However, we introduced a method of selecting the highest SUVR value among each target region as the cutoff value, rather than the SUVR value from global regions, and carefully examined whether this can be better matched with visual findings in clinical practice. If amyloid PET is performed under conditions similar to those in our institution, it is important to note that if the SUVR of any target regions is higher than our cutoff value, it is highly likely to be a positive finding for amyloid deposition. Future related studies are warranted to yield stronger evidence.

We used the cerebellar grey matter as a reference region for quantitative analysis of amyloid PET images. Regarding ^18^F-flutemetamol, the activity of the pons is used as the standard for visual reading. Accordingly, previous studies have used the pons as a reference for quantitative analysis of PET images^17–19^. As the cerebellum has been used as a reference region in various brain PET images as well as amyloid PET analysis ^20–22^, our institution used the cerebellar grey matter as a reference region for ^18^F-flutemetamol. Further analysis of our results using the pons as a reference showed that Heuron yielded higher overall SUVRs than did PMOD (0.61 ± 0.14 vs. 0.57 ± 0.13, *p* < 0.001, supplementary Table 1); moreover, Bland–Altman plot analysis indicated that PMOD and Heuron could be used interchangeably (Supplementary Fig. 1). Additionally, ROC curve analysis revealed no between-method difference in the diagnostic performance for visual positivity of amyloid deposition (Supplementary Fig. 2). With the pons as a reference region, the cutoff SUVR value for distinguishing visual positivity of amyloid deposition was 0.65 for Heuron and 0.64 for PMOD, which is relatively consistent with previous reports^17,18^. Those who use the pons as a reference area should refer to the supplementary results.

Here, we would like to discuss the advantages and disadvantages of Heuron over PMOD. First, Heuron has a much shorter analysis time, compared with PMOD. Specifically, we found that the analysis time for PMOD and Heuron was > 10 min and 3–5 min, respectively; however, the analysis time may vary according to the computer hardware specifications. Second, as PMOD only provides standardized uptake value (SUV) data for each brain region, for research purposes, the user is required to perform the extra step of dividing these values by the reference SUV to obtain the SUVR. Contrastingly, Heuron provides both SUV data and SUVR values for target regions, which renders it more convenient for clinical use. Additionally, Heuron provides reference settings for the whole cerebellum, cerebellar grey matter, and pons, which can be simply selected by users. Finally, PMOD allows quantification analysis without magnetic resonance (MR) data; contrastingly, Heuron requires MR brain images for PET image analysis. However, it is important to note that quantitative analysis of amyloid PET without MR data in PMOD may exhibit very low accuracy.

This study has several limitations. First, ^18^F-flutemetamol was the only radiopharmaceutical agent used for amyloid PET. Other institutions may have preferences for other commercially available ^18^F-labeled radiopharmaceuticals, including ^18^F-florbetaben, ^18^F-florbetapir, and ^18^F-florapronol, for PET imaging of amyloid deposition^23,24^. Further studies are required to compare the quantification values obtained using Heuron and PMOD with radiopharmaceuticals other than ^18^F-flutemtamol. Second, we only compared Heuron with PMOD. As aforementioned, other tools are clinically used for amyloid PET quantification, including *MIMneuro*, *CortexID*, and *Syngo.VIA*^9^. Therefore, future studies are required to compare Heuron with software other than PMOD. Third, we did not include data on the entire cerebellum, including its white matter. Previous studies have used the whole cerebellum as a reference area for quantitative analysis of amyloid PET, along with the cerebellar grey matter and pons^17,18^. The preferred reference region may differ across institutions. As mentioned earlier, we could not analyse data using other brain regions, because the whole cerebellum was not used as a reference in our institution. Future studies that include data obtained from the whole cerebellum are necessary. Fourth, we did not perform a comparative analysis of the volume of each brain region. Specifically, we only analysed SUVR values obtained from PET images and did not consider volume data. As both PMOD and Heuron provide data regarding volume by area, future studies are required to compare the volume values yielded by both software packages. Finally, this study did not include the final clinical diagnosis of the patient. Amyloid PET is only an auxiliary tool for AD diagnosis and not all positive results on amyloid PET are indicative of an AD diagnosis^25^. Accordingly, we focused on the diagnostic performance of the visual positivity of amyloid deposition rather than the diagnosis of AD. Nonetheless, our findings may inform the detection of the presence or absence of amyloid deposits on amyloid PET images in clinical practice.

In conclusion, Heuron and PMOD showed comparable diagnostic performance in evaluating visual positivity for amyloid deposition; moreover, they can be used interchangeably.

## Methods

### Participants

This study included 408 patients (age, 72.0 ± 7.3 years, female/male = 286/122) with ^18^F-flutemetamol PET and 3D T1-weighted brain MR images (mean time interval between the two imaging sessions, 8.5 ± 7.3 days) available for quantitative amyloid analysis. The imaging data used in this study were provided by the Biobank of Ajou University Hospital, a member of the Korea Biobank Network. Participants were voluntarily recruited from individuals who visited neurology or psychiatric memory outpatient clinics. This study was approved by the Institutional Review Board of Ajou University Hospital (AJOUIRB-EX-2023-020). Written informed consent was obtained from all the participants.

### Brain PET/CT acquisition

PET/computed tomography (CT) data were acquired on a Discovery ST scanner (GE Healthcare, Milwaukee, WI, USA). Additionally, ^18^F-flutemetamol was purchased from GE Healthcare (Vizamyl, GE Healthcare, Seoul, South Korea). Radiochemical purity was confirmed, and the specific activity at the end of the synthesis was satisfactory for use in PET imaging before daily use. The patients were intravenously injected with ^18^F-flutemetamol (mean dose: 204.48 [± 9.13] MBq) and allowed to rest for 90 min. Brain PET data (20 min [4 × 5 min frames], 3D mode) were obtained after brain CT (100 kV, 95 mA; section width, 3.75 mm). The PET images were iteratively reconstructed (i.e., ordered subsets of expectation maximisation with two iterations and 21 subsets, Gaussian filter [full width at half maximum = 2.14 mm], with a 128 × 128 matrix), and the CT data was used for attenuation correction.

### Visual analysis of PET images

The ^18^F-flutemetamol PET images were visually evaluated by a single nuclear medicine specialist (Y.S.A.) blinded to the patient’s clinical information. This specialist is highly trained in the visual interpretation of amyloid PET images, has completed the reader training program provided by GE Healthcare^8^, and has experience in reading > 1000 ^18^F-flutemetamol PET images.

For visual analysis, transverse, sagittal, and coronal PET images were displayed on an Advantage Workstation (AW VolumeShare 2; GE Healthcare), with the scale set at 90% intensity for the pons. Next, the specialist visually evaluated whether amyloid deposits were present in the target sites of ^18^F-flutemetamol PET imaging including the frontal, parietal, and temporal lobe; posterior cingulate/precuneus (PC/PCC); and striatum. Cases where amyloid deposition in any of these were visually detected were interpreted as ‘positive’.

### Quantitative analysis of PET image using PMOD

Quantitative analysis of PET images was performed using the Maximum Probability Atlas application in PMOD Neuro Tool (version 3.802, PMOD Technologies Ltd., Zurich, Switzerland). First, the static PET image series was loaded, and the individual grey matter probability map was calculated by segmenting the T1-weighted MR image of each patient. The brain was split into the left and right hemispheres and the cerebellum. The MR images were spatially normalised to the Montreal Neurological Institute T1 template. The segmented and normalised MR images were rigidly matched to the PET images; further, their alignments were visually checked by a nuclear medicine specialist with 14 years of experience in brain PET (Y.S.A.). The automated anatomic labelling (AAL) volumes-of-interest (VOIs) atlas^26^ was transformed into the MR space; moreover, cortical structures were intersected with the grey matter probability map (mask threshold = 0.3). The final VOIs applied to the matched PET series to calculate the average regional uptake, which was represented as the SUV, were determined based on the radioactivity injection dosage and body weight. The VOIs of the target regions, including the frontal, PC/PCC, parietal, temporal, and striatum regions, were selected^27^. The average SUVs obtained from each brain region were divided by the average cerebellar grey matter SUV to obtain the SUVR.

Table 1 shows the detailed structures constituting each brain area, and Fig. 4 shows the representative outline contours of the VOIs for the selected areas.

**Table 1:** The structures included in each brain region.

	Frontal lobe	Precuneus/posterior cingulate	Parietal lobe	Temporal lobe	Striatum	Cerebellum
PMOD	Precentral gyrus Superior frontal gyrus Middle frontal gyrus Inferior frontal gyrus Supplementary motor area Olfactory cortex Paracentral lobule	Posterior cingulate gyrus Precuneus	Postcentral gyrus Superior parietal gyrus Inferior parietal gyrus Supramarginal gyrus Angular gyrus	Superior temporal gyrus Middle temporal gyrus Inferior temporal gyrus	Caudate nucleus Putamen	Cerebellum crus Cerebellum
Heuron	Caudal middle frontal gyrus Lateral orbitofrontal gyrus Medial orbitofrontal gyrus Paracentral lobule Pars opercularis Pars orbitalis Pars triangularis Precentral gyrus Rostral middle frontal gyrus Superior frontal gyrus	Posterior cingulate gyrus Precuneus	Inferior parietal lobule Postcentral gyrus Superior parietal lobule Supramarginal gyrus	Entorhinal cortex Fusiform gyrus Inferior temporal gyrus Middle temporal gyrus Parahippocampal gyrus Superior temporal gyrus Transverse temporal gyrus	Caudate nucleus Putamen	Cerebellum-cortex

### Quantitative analysis of PET images using Heuron

Heuron Beta Amyloid (Heuron Co., Ltd., Republic of Korea, iheuron.com) allows automatic quantification of PET tracer binding. Briefly, it uses 3D brain MR and PET images as input to provide SUVR information based on the voxel image intensity of segmented brain regions. Moreover, the segmentation engine provides a deep learning architecture for segmenting the entire brain into 97 ROIs (including background regions such as surrounding non-brain tissues) within 90 s on the graphics processing unit^28^. This architecture is inspired by QuickNAT, which is a convolutional neural network^29^. It is trained using MR images and manually annotated labels. It is composed of three fully convolutional neural networks operating on axial, coronal, and sagittal slices; further, it involves aggregation steps to infer the final segmentation (Supplementary Fig. 3). The PET image was co-registered to the individual’s MRI, followed by calculation of the SUVs of the brain regions in the native MRI space. We selected the frontal, PC/PCC, parietal, temporal, and striatal regions as the target regions and included cerebellar grey matter as the reference region. The SUVR of each target region was obtained by dividing the mean SUV by the mean SUV of the cerebellar grey matter. Table 1 and Fig. 4 present details of the VOIs.

### Statistical analysis

All statistical analyses were performed using MedCalc software (version 20.116; MedCalc Software Ltd., Ostend, Belgium). The required sample size was calculated using a significance (α) level of 5% and statistical power (1−β) of 80%, with the number of included participants (*n* = 408) meeting the sample size requirements.

The normality of data distribution was assessed using the Kolmogorov–Smirnov test. Continuous variables are expressed as means and SD, and appropriate parametric statistical methods were used. SUVRs obtained from each software tool were compared using paired-sample t-tests. Additionally, the two quantification software methods were graphically compared using Bland–Altman plot analysis. The maximum allowed difference was defined using the following Eq. ^30^; (coefficient of variation [CV]^2^_PMOD_ + CV^2^_Heuron_)^1/2^. An independent samples t-test and chi-square test were used to analyse differences in age and sex, respectively, between patients with and without visual amyloid deposition. The diagnostic performance of the obtained SUVRs in evaluating the presence or absence of amyloid deposition was examined through ROC curve analysis; additionally, cutoff values based on the Youden index^31^ were obtained. The highest SUVR value among those obtained from the frontal, PC/PCC, parietal, temporal, and striatal regions was included in the ROC curve analysis. Finally, we compared the AUC values for each tool. Statistical significance was set at *p* < 0.05.

### Ethics declarations

This study was conducted in accordance with the guidelines of the Declaration of Helsinki and approved by the Institutional Review Board of Ajou University (approval number: AJOUIRB-EX-2023-020). Written informed consent was obtained from all participants.

## Supplementary Information

Supplementary Figures.

Supplementary Table S1.

## Data Availability

The datasets used and/or analysed during the current study are available from the corresponding author on reasonable request.
