# Supplementary material for: Comparison of automated quantification of amyloid deposition between PMOD and Heuron
Source: Sci Rep. 2023 Jun 19;13:9891. doi: 10.1038/s41598-023-36986-5 (PMC10279744; doi:10.1038/s41598-023-36986-5)
Supplement: Supplementary file 1 — Supplementary Figures. [file 41598_2023_36986_MOESM1_ESM.docx]

**Comparison of automated quantification of amyloid deposition between PMOD and Heuron**

Hyun Woong Roh^1^^†^, Sang Joon Son^1†^, Chang Hyung Hong^1†^, So Young Moon^2^, Sun Min Lee^2^, Sang Won Seo^3^, Seong Hye Choi^4^, Eun-Joo Kim^5^, Soo Hyun Cho^6^, Byeong Chae Kim^6^, Seongbeom Park^7^, Soohwa Song^7^, Young-Sil An^8*^,

*^1^Department of Psychiatry Ajou University School of Medicine, Suwon, Korea, ^2^Department of Neurology, Ajou University School of Medicine, Suwon, Korea, ^3^Department of Neurology, Samsung Medical Center, Sungkyunkwan University School of Medicine, Seoul, Korea, ^4^Department of Neurology, Inha University School of Medicine, Incheon, Korea, ^5^Department of Neurology, Pusan National University Hospital, Pusan National University School of Medicine and Medical Research Institute, Busan, Korea, ^6^Department of Neurology, Chonnam National University Medical School, Chonnam National University Hospital, Gwangju, Korea, ^7^Heuron Co., Ltd., Incheon, Korea, ^8^Department of Nuclear Medicine and Molecular Imaging, Ajou University School of Medicine, Suwon, Korea*

**†**These authors contributed equally to this work

**^*^Corresponding author:**

Young-Sil An, Professor

Department of Nuclear Medicine and Molecular Imaging, School of Medicine, Ajou University, 206, World cup-ro, Yeongtong-gu, Suwon-si, Gyeonggi-do, Suwon, Korea 16499

Phone: +82-31-219-5947

Fax: +82-31-219-5950

E-mail: [aysays77@naver.com](mailto:aysays77@naver.com)


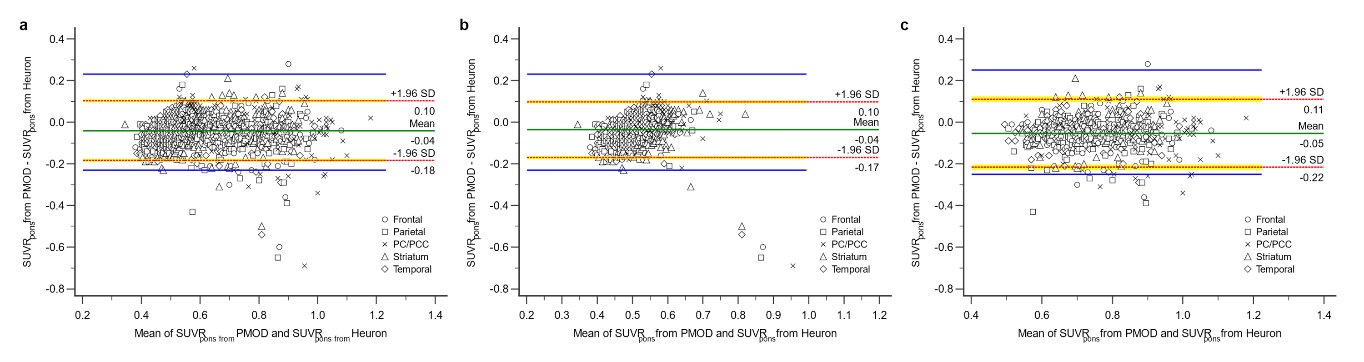


**Supplementary Figure 1.** Scatter diagrams of the differences plotted against the averages of SUVR values obtained using the PMOD and Heuron, with the pons as the reference region. Graph ‘a’ presents the result from all participants, and the other graphs present the results divided into the visually amyloid-positive (b) and amyloid-negative groups (c). The mean difference value according to Bland–Altman plot analysis is drawn as a green horizontal line in the graph. The limits of agreement (LoA) representing the mean difference ± 1.96 standard deviations (SD) are indicated by red horizontal dotted lines. The partially yellow lines show the 95% confidence interval of the LoA. Blue horizontal lines represent the maximum allowed between-method difference, which was not exceeded as indicated by the LoAs (red horizontal dotted lines) in all graphs (a, b, and c). Therefore, SUVR values yielded by PMOD and Heuron could be interchangeably used.


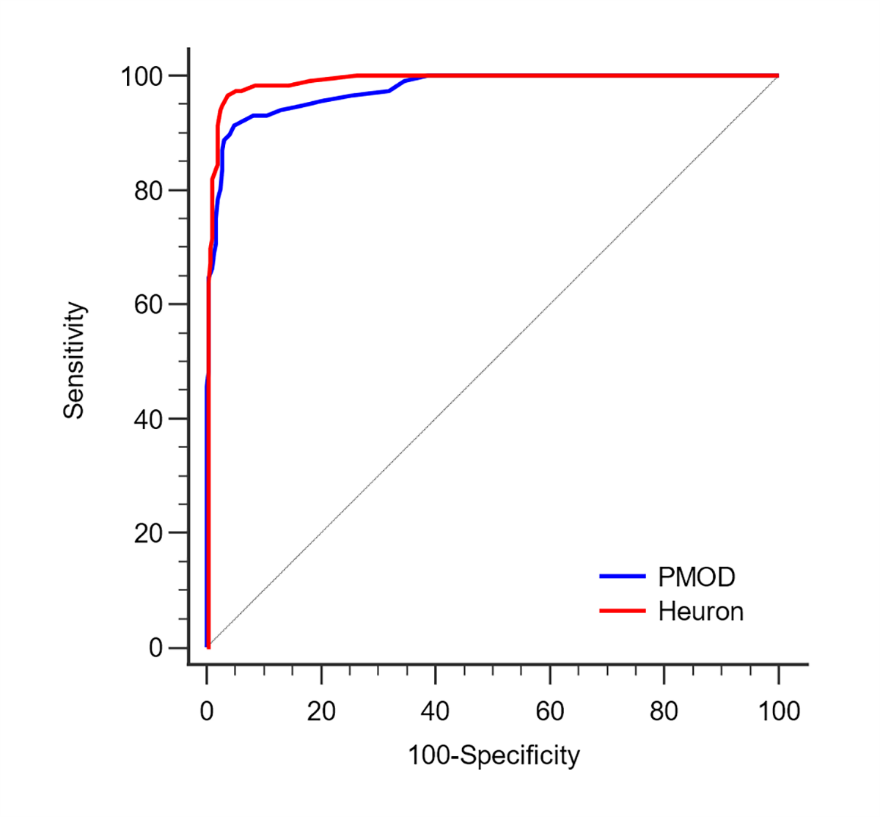


**Supplementary Figure 2.** Receiver Operating Characteristic (ROC) curves of SUVR values for assessing visual positivity of amyloid deposition, with the pons as the reference region. There was no significant difference in the diagnostic performance in detecting the visual presence or absence of amyloid deposits in PET images between PMOD (area under the ROC curve [AUC] = 0.975; cut-off value = 0.64; sensitivity = 91.4%; specificity = 95.2%) and Heuron (AUC = 0.989; cut-off value = 0.65; sensitivity = 96.6%; specificity = 96.2%; *p* = 0.062).


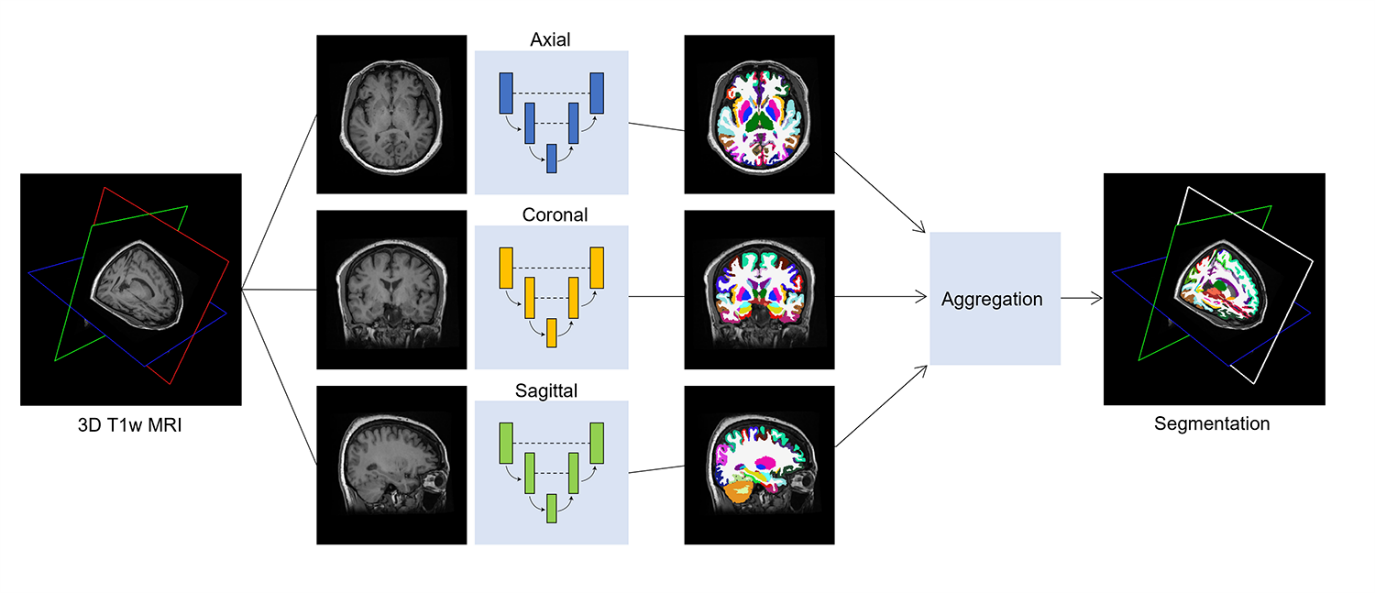


**Supplementary Figure 3.** Final view aggregation step in Heuron. Each network learns an anatomical representation of brain structures within the coronal, axial, or sagittal views, depending on the orientation of the two-dimensional (2D) slices. The final segmentation is generated by combining all three networks.
