# Supplementary material for: Comparison of automated quantification of amyloid deposition between PMOD and Heuron
Source: Sci Rep. 2023 Jun 19;13:9891. doi: 10.1038/s41598-023-36986-5 (PMC10279744; doi:10.1038/s41598-023-36986-5)
Supplement: Supplementary file 2 — Supplementary Table S1. [file 41598_2023_36986_MOESM2_ESM.docx]

**Comparison of automated quantification of amyloid deposition between PMOD and Heuron**

Hyun Woong Roh^1^^†^, Sang Joon Son^1†^, Chang Hyung Hong^1†^, So Young Moon^2^, Sun Min Lee^2^, Sang Won Seo^3^, Seong Hye Choi^4^, Eun-Joo Kim^5^, Soo Hyun Cho^6^, Byeong Chae Kim^6^, Seongbeom Park^7^, Soohwa Song^7^, Young-Sil An^8*^,

*^1^Department of Psychiatry Ajou University School of Medicine, Suwon, Korea, ^2^Department of Neurology, Ajou University School of Medicine, Suwon, Korea, ^3^Department of Neurology, Samsung Medical Center, Sungkyunkwan University School of Medicine, Seoul, Korea, ^4^Department of Neurology, Inha University School of Medicine, Incheon, Korea, ^5^Department of Neurology, Pusan National University Hospital, Pusan National University School of Medicine and Medical Research Institute, Busan, Korea, ^6^Department of Neurology, Chonnam National University Medical School, Chonnam National University Hospital, Gwangju, Korea, ^7^Heuron Co., Ltd., Incheon, Korea, ^8^Department of Nuclear Medicine and Molecular Imaging, Ajou University School of Medicine, Suwon, Korea*

**†**These authors contributed equally to this work

**^*^Corresponding author:**

Young-Sil An, Professor

Department of Nuclear Medicine and Molecular Imaging, School of Medicine, Ajou University, 206, World cup-ro, Yeongtong-gu, Suwon-si, Gyeonggi-do, Suwon, Korea 16499

Phone: +82-31-219-5947

Fax: +82-31-219-5950

E-mail: [aysays77@naver.com](mailto:aysays77@naver.com)

**Supplementary Table 1**. Comparison of SUVRs from pons reference between PMOD and Heuron

| Brain region | SUVR based on pons reference by PMOD (mean ± SD^*^) | SUVR based on pons reference by Heuron (mean ± SD) | *p*-value^†^ |
| --- | --- | --- | --- |
| Frontal | 0.56 ± 0.13 | 0.60 ± 0.14 | *p* < 0.001 |
| PC/PCC^‡^ | 0.62 ± 0.16 | 0.64 ± 0.18 | *p* < 0.001 |
| Parietal | 0.54 ± 0.14 | 0.60 ± 0.14 | *p* < 0.001 |
| Temporal | 0.53 ± 0.12 | 0.57 ± 0.13 | *p* < 0.001 |
| Striatum | 0.58 ± 0.13 | 0.62 ± 0.11 | *p* < 0.001 |
| Total | 0.61 ± 0.14 | 0.57 ± 0.13 | *p* < 0.001 |

^*^Standard deviation, ^†^*p*-value from the paired samples t-test, ^‡^ posterior cingulate/precuneus
